# Nanomaterial-based strategies overcome PD-1 related intrinsic immune resistance

**DOI:** 10.20517/cdr.2025.207

**Published:** 2026-04-21

**Authors:** Yiyang Lin, Jianliang Shen

**Affiliations:** ^1^Department of Bioengineering, Northeastern University, Boston, MA 02108, USA.; ^2^Wenzhou Institute, University of Chinese Academy of Sciences, Wenzhou 325001, Zhejiang, China.; ^3^National Engineering Research Center of Ophthalmology and Optometry, Eye Hospital, Wenzhou Medical University, Wenzhou 325027, Zhejiang, China.

**Keywords:** PD-1/PD-L1, immunotherapy, immune resistance, nanomaterials

## Abstract

Immune-checkpoint inhibitors targeting programmed cell death protein 1 (PD-1) or programmed death-ligand 1 (PD-L1) have substantially improved outcomes for patients with multiple cancer types; however, primary (intrinsic) resistance remains common and limits durable responses. Mechanistically, such resistance can arise from impaired interferon-γ signaling (including Janus kinases-signal transducer and activator of transcription dysfunction), tumor-intrinsic oncogenic pathway alterations [e.g., phosphatase and tensin homolog (PTEN) loss with downstream phosphoinositide 3-kinase/protein kinase B hyperactivation and Wnt/β-catenin-associated immune escape], and tumor-extrinsic immunosuppression mediated by PD-L1-upregulated suppressive myeloid populations such as myeloid-derived suppressor cells. These pathways converge on reduced T-cell effector function, compromised immune recognition, and reinforcement of an immunosuppressive tumor microenvironment (TME), collectively diminishing the clinical benefit of PD-1/PD-L1 blockade. In this review, we synthesize current evidence on primary (intrinsic) resistance to PD-1/PD-L1 blockade and discuss how nanomaterial-enabled interventions can be mechanistically matched to these resistance determinants. The nanotechnology-based therapeutic strategies were classified as four categories: (i) modulation of resistance-associated signaling pathways; (ii) direct blockade/interception of the PD-1/PD-L1 axis; (iii) immune-checkpoint gene silencing; and (iv) TME reprogramming.

## INTRODUCTION

Cancer immunotherapy has become an integral component of modern oncology. Immune checkpoint inhibitors (ICIs) have demonstrated substantial clinical benefits by reinvigorating antitumor T-cell responses, enabling durable tumor regression and long-term survival in a subset of patients across multiple malignancies^[1-3]^. Despite these advances, important limitations continue to hinder the broader and more consistent success of immunotherapy. Only a minority of patients derive sustained benefit, while many exhibit primary resistance or develop acquired resistance after an initial response^[4,5]^. Overall, immunotherapy represents a highly effective yet still constrained strategy for cancer treatment.

Among immune checkpoint pathways, the programmed cell death protein 1 (PD-1) and programmed death-ligand 1 (PD-L1) axis is one of the most extensively validated therapeutic targets and has become a cornerstone of ICI therapy. In this pathway, PD-1 expressed on activated T cells binds to PD-L1 on tumor cells and other components of the tumor microenvironment (TME), thereby attenuating T-cell activation and facilitating immune evasion. Although PD-1/PD-L1 blockade can restore antitumor immunity in some patients, therapeutic resistance remains common^[6]^. Notably, primary (intrinsic) resistance refers to a lack of clinical response at the beginning of PD-1/PD-L1 inhibition.

To address these limitations, nanomaterial-based platforms offer a promising avenue. Targeted nano-drug delivery systems can enhance tumor accumulation of immunotherapeutic agents, enable controlled or stimuli-responsive release, and facilitate co-delivery of small molecules and nucleic acids [e.g., small interfering RNA (siRNA)] to modulate resistance-associated pathways^[7,8]^. Accordingly, this review focuses on nanomaterial-based strategies for overcoming primary resistance to PD-1/PD-L1 blockade. It highlights mechanistically guided approaches, including signaling pathway modulation, checkpoint gene silencing, localized PD-1/PD-L1 interception, and TME reprogramming, all of which may help broaden and prolong the clinical benefits of checkpoint immunotherapy.

## FUNCTION OF THE PD-1/PD-L1 PATHWAY

### PD-1

PD-1 (CD279) is an inhibitory receptor of the CD28/cytotoxic T-lymphocyte–associated protein 4 (CTLA-4) family that was initially identified as a gene upregulated during programmed cell death^[9]^. PD-1 is widely expressed on activated T cells and is also inducible on B cells, monocytes, macrophages, and other immune subsets^[10]^. In cancer, PD-1 functions as a key immune checkpoint that contributes to T-cell dysfunction/exhaustion, and its upregulation on tumor-reactive T cells is associated with reduced antitumor effector function.

### PD-L1

PD-L1 (B7-H1, CD274) is the first ligand of PD-1^[11]^. It is mainly distributed in resting lymphocytes, antigen-presenting cells (APCs), and some types of tumor cells^[12,13]^. In cancer, PD-L1 expression is used as a biomarker for PD-1/PD-L1 blockade and can mediate immune escape by suppressing T-cell activity at the tumor-immune interface^[13]^.

### The pathway of PD-1 and PD-L1

The interaction between PD-1 and PD-L1 plays a fundamental role in regulating T-cell recognition of tumor cells and contributes to immune evasion through distinct signaling pathways^[14]^. This axis is central to the interplay between the host immune defense system and tumor cells. The binding of PD-1 to PD-L1 suppresses T-cell activation by inhibiting T-cell receptor (TCR) signaling and downregulating TCR-mediated lymphocyte proliferation. The PD-1/PD-L1 pathway helps maintain immune homeostasis by limiting excessive immune activation during infection and inflammation, but tumors can exploit this pathway to suppress antitumor immunity^[15]^. As a result, tumor cells are able to evade immune effector responses. One of the key mechanisms underlying this process is the overexpression of PD-L1 on tumor cells, which increases the likelihood of PD-1/PD-L1 binding. This interaction acts as a barrier between T cells and tumor cells, leading to immune suppression and facilitating tumor immune evasion^[16]^. The PD-1/PD-L1 interaction mainly occurs at the immunological synapse, which is the cell-cell contact interface on the plasma membrane between a PD-1^+^ T cell and a PD-L1^+^ APC or target/tumor cell^[17]^. Given the mechanisms described above, regulating the PD-1/PD-L1 signaling pathway and controlling the expression levels of PD-1 and PD-L1 are critical aspects in cancer therapy.

## PRIMARY IMMUNE RESISTANCE TO PD-1/PD-L1

**Figure 1:**
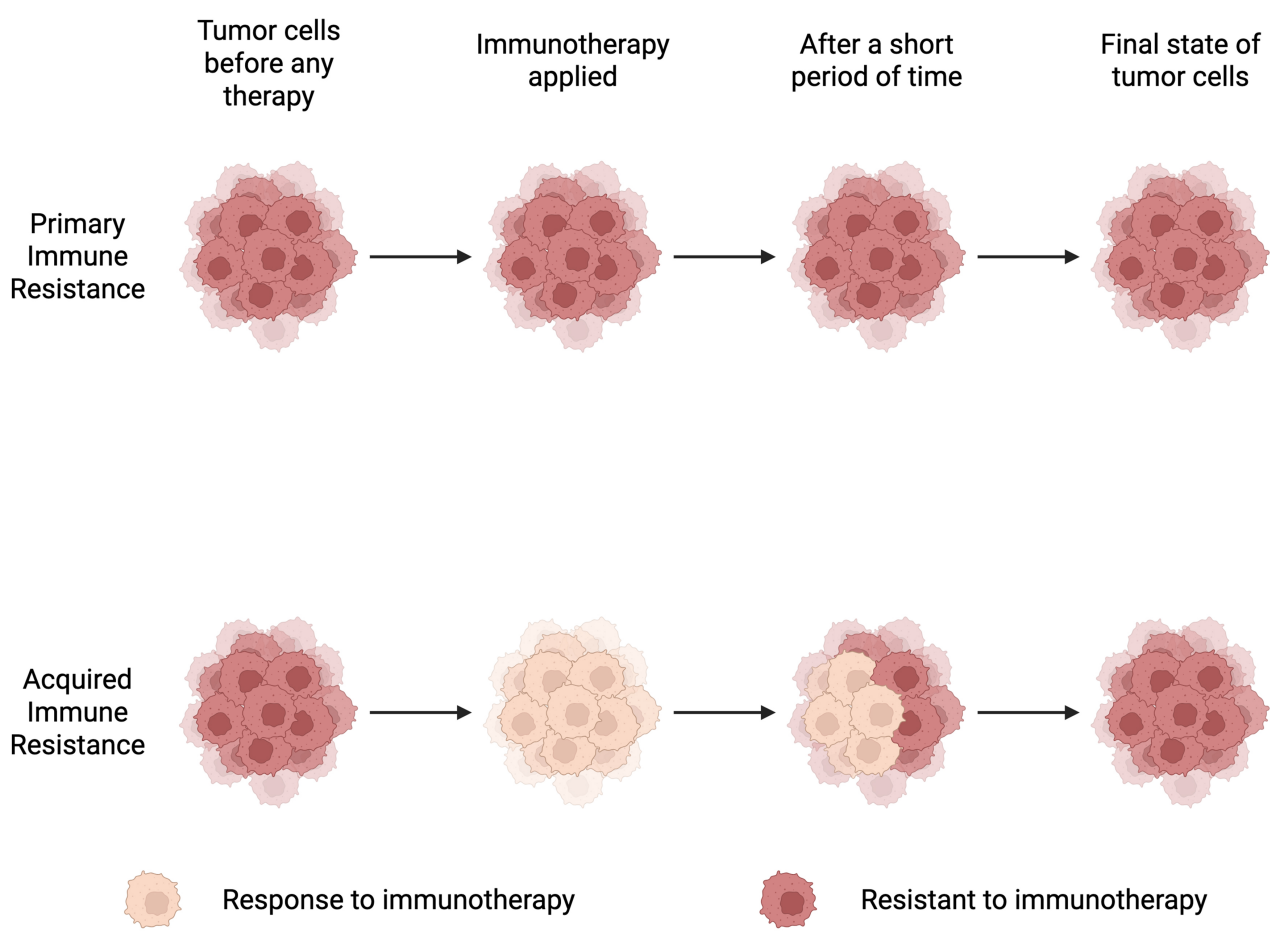
Primary and acquired immune resistance. This diagram illustrates two patterns of resistance to immunotherapy: primary (intrinsic) resistance, in which tumors do not respond from the start of therapy, and acquired resistance, in which tumors initially respond but later progress after a period of treatment. Created in BioRender. Lin, Y. (2026) https://BioRender.com/xnemqkt.

Immune resistance mainly falls into two categories: primary resistance and acquired resistance. Primary resistance, also referred to as intrinsic resistance, refers to a lack of clinical response from the beginning of treatment, whereas acquired resistance describes disease progression after an initial response^[18]^. Although PD-1/PD-L1 blockade immunotherapy is generally more effective and durable than other treatment modalities, clinical evidence indicates that primary/tumor-intrinsic resistance is common and remains a major barrier to broader clinical success^[19]^. Therefore, strategies to reduce the occurrence of primary resistance are essential for improving therapeutic outcomes. Studies have shown that the underlying causes of immune resistance can be broadly classified into tumor-intrinsic and tumor-extrinsic factors^[18]^. Tumor-intrinsic factors arise from alterations within tumor cells. In contrast, tumor-extrinsic factors originate outside tumor cells, primarily within the TME. Intrinsic mechanisms involve disruptions in antitumor immune signaling, aberrant activation or suppression of intracellular pathways in tumor cells, and additional tumor-intrinsic alterations that collectively foster an immunosuppressive state^[20]^. Extrinsic factors involve the components of the local TME, such as regulatory T cells (Tregs), myeloid-derived suppressor cells (MDSCs), and other inhibitory immune checkpoints, which support tumor growth and immune evasion^[20]^. The following paragraph discusses the tumor-intrinsic mechanisms that contribute to primary resistance to PD-1/PD-L1 blockade [Figure 1].

### Tumor-extrinsic

#### MDSCs

MDSCs are a heterogeneous population of immature myeloid cells derived from the bone marrow, known for their potent immunosuppressive activity within the TME^[21]^. Using flow cytometry, Ruan *et al.* found that PD-L1 is involved in the immune function of human MDSCs, indicating that these PD-L1^+^ cells may suppress T cells through PD-1/PD-L1 interactions and the overexpression of PD-1^[22]^. This finding suggests that MDSCs express PD-L1 on their surface. In the research of Lu *et al*., around 60% of total MDSCs in the cancer patients are PD-L1^+^ MDSCs, and the proportion of PD-L1^+^ MDSCs was higher in blood from colon cancer patients than healthy people^[23]^. Under hypoxic conditions in the TME, as well as in the presence of inflammatory cytokines, the PD-1/PD-L1 axis is upregulated in both intertumoral and circulating MDSCs^[24]^. During inflammation, MDSCs also release a protein known as S100A9, which can activate signaling pathways such as extracellular signal-regulated kinase 1/2 (ERK1/2) in MDSCs, thereby upregulating PD-L1 expression and further promoting immunosuppression^[25]^.

### Tumor-intrinsic

#### Interferon-γ pathway (STAT3, JAK1 and JAK2)

Interferon-γ (IFN-γ), a cytokine secreted by activated T cells and APCs, functions as a key immunomodulatory messenger. The IFN-γ receptor (IFNGR) consists of two subunits, IFNGR1 and IFNGR2^[26]^. Their signals are initiated by binding to the IFNGR, leading to activation of Janus kinases (JAK1 and JAK2). Subsequent phosphorylation events recruit and activate signal transducer and activator of transcription (STAT) family transcription factors, which then translocate to the nucleus to regulate multiple downstream gene expression programs^[27]^. It contributes to immune evasion by upregulating PD-L1 expression on tumor cell surfaces^[28]^. Intrinsic signaling through PD-L1 leads to the inhibition of STAT3, which in turn suppresses IFN-induced apoptotic pathways in tumor cells^[29]^. In the study reported by Doi *et al.*, treatment with the JAK2 inhibitor AG490 markedly reduced PD-L1 expression by suppressing its transcriptional induction and subsequent protein synthesis^[30]^. Another study in colorectal cancer xenograft models shows that the overexpression of fibroblast growth factor receptor 2 (FGFR2) can promote PD-L1 expression and tumor growth, which may be blocked by downregulation of the JAK/STAT3 pathway^[31]^. Together, these studies suggest that IFN-γ can contribute to intrinsic immune resistance by modulating PD-L1 expression. Although IFN-γ signaling induces PD-L1 expression, which suppresses T-cell effector function and facilitates immune escape, under PD-1/PD-L1 blockade this induction often serves as a sign of a T-cell-inflamed microenvironment and correlates with improved therapeutic response. Conversely, disruption of IFN-γ pathway components impairs antigen presentation and T-cell recognition, thereby compromising antitumor immunity. One important mechanism of primary resistance involves tumor cells altering or downregulate IFN-γ signaling through loss-of-function mutations in JAK1/2, thereby inhibiting PD-1/PD-L1 blockade^[20]^. Although tumor cells remain detectable by T cells in the presence of JAK1/2 loss-of-function mutations, JAK1/2 mutations against the antiproliferative effects of IFN-γ and abrogate IFN-γ-induced upregulation of PD-L1 on their surface^[32]^. Such mutations are therefore considered a major cause for the primary resistance in IFN-γ. With loss-of-function mutations in JAK1/2, antitumor T cells have a reduced ability to recognize and kill cancer cells, indicating that these mutations can impair tumor immune responses^[33]^. In addition, loss-of-function mutations exert another pivotal effect by preventing IFN-γ-induced PD-L1 expression, thereby rendering PD-1/PD-L1 blockade ineffective^[33]^. Meanwhile, the genetic mutations can also contribute to immune resistance in IFN-γ. For example, in a clustered regularly interspaced short palindromic repeats (CRISPR) screen, the alteration of PD-1/PD-L1 by the resistance of IFN-γ will cause the mutation of tyrosine-protein phosphatase non-receptor type 2 (Ptpn2), which attenuates JAK1 and STAT1 signaling^[34]^.

#### Wnt/β-catenin pathway

The Wnt/β-catenin pathway is frequently activated in diverse tumor types. Wnt/β-catenin signaling is an evolutionarily conserved pathway that regulates processes ranging from embryogenesis to oncogenesis and contributes to tumor progression by promoting resistance to immune-mediated oncolysis^[27,35]^. Canonical Wnt/β-catenin signaling is initiated when a Wnt ligand binds to cell-surface receptors, triggering downstream signal transduction that culminates in the nuclear translocation of β-catenin and transcriptional activation^[35]^. In melanoma, approximately one-third of specimens with elevated Wnt/β-catenin activity exhibit a marked absence of T-cell infiltration, suggesting that activation of Wnt/β-catenin signaling may impede the priming of *de novo* antitumor immune responses^[35]^. Abnormal Wnt/β-catenin signaling facilitates malignant transformation and contributes to resistance to cancer therapy^[36]^. Several representative examples are summarized below. In melanoma, the main reasons for anti-PD-L1 resistance induced by the Wnt/β-catenin pathway include the lack of a T-cell genomic signature and reduced T-cell infiltration^[37]^. In triple-negative breast cancer (TNBC), exploiting the interplay between Wnt signaling and PD-L1 expression allows selective Wnt pathway inhibition to suppress PD-L1 or pathway activation to enhance its expression for therapeutic purposes^[38]^. The Wnt/β-catenin pathway in fibroblasts acts as an inducer of the upregulation of PD-L1 expression^[39]^. In the study of Wang *et al.*, PD-L1 and p-β-catenin were positively correlated in nasopharyngeal carcinoma (NPC)^[40]^. The influence of Wnt/β-catenin signaling on PD-L1 upregulation underscores its pivotal role in tumor immunity^[41]^. Taken together, these findings suggest that tumor-intrinsic β-catenin activation may represent one mechanism of primary resistance to immunotherapy. Given the broad involvement of Wnt/β-catenin signaling in tumor biology, this pathway may be an important contributor to intrinsic resistance to PD-1/PD-L1 blockade.

#### PTEN loss and PI3K/AKT pathway

PTEN, a well-known tumor suppressor, plays a central role in the phosphoinositide 3-kinase (PI3K)/protein kinase B (AKT) signaling pathway (PI3K/AKT pathway)^[42]^. The loss of PTEN will trigger the activation of PI3K/AKT signaling pathway, facilitate the overexpression of PD-L1, and is associated with tumor immune escape in many types of cancers, including hepatocellular carcinoma, prostate cancer, and breast cancer^[42-47]^. PI3K/AKT pathway attenuates T-cell-mediated tumor cytotoxicity, increases PD-L1 expression, and ultimately fosters the development of resistance to PD-1 inhibitors^[48-50]^. In the study of Peng *et al.*, loss of PTEN was also shown to contribute to primary resistance to anti-PD-1 therapy^[49]^. Moreover, accumulating evidence suggests PI3K/AKT pathway is possible to induce intrinsic resistance to immune therapy through the relation with PD-1/PD-L1. Representative examples are summarized below. Given that PTEN negatively regulates PI3K, inhibition of PI3K has been proposed as a therapeutic strategy to enhance antitumor immunity^[27]^. In murine models, treatment with a selective PI3Kβ inhibitor was found to enhance the efficacy of anti-PD-1 antibodies^[49]^. Zhao *et al.* reported that the PI3K/AKT/mechanistic target of rapamycin (mTOR) pathway in gastrointestinal stromal tumors (GIST) can modulate PD-1/PD-L1 signaling and reduce apoptosis of CD8^+^ T cells^[51]^. Analyses of glioblastoma tissue specimens have demonstrated that T cells more efficiently lyse tumor cells with wild-type PTEN than those bearing PTEN mutations, and this impaired cytotoxicity correlates with upregulated expression of the B7-H1 receptor^[50]^. Furthermore, activation of the PI3K-AKT pathway in triple-negative breast cancer MDA-MB-231 cells led to increased PD-L1 expression, whereas treatment with the PI3K inhibitor LY294002 resulted in its downregulation^[52,53]^. Collectively, these studies reveal a close relationshipbetween the PTEN/PI3K-AKT axis and PD-1/PD-L1 signaling, suggesting that this pathway may contribute to intrinsic resistance to PD-1/PD-L1-targeted immunotherapy.

**Figure 2:**
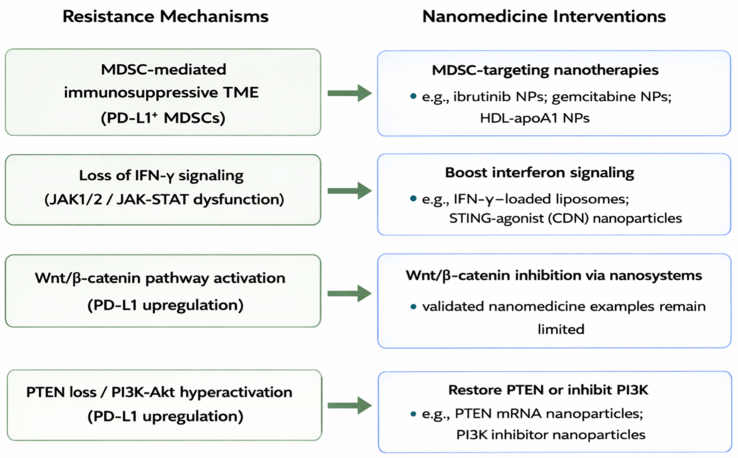
The mechanistic map linking resistance mechanisms to nano-interventions. MDSC: Myeloid-derived suppressor cell; TME: tumor microenvironment; PD-L1: programmed death-ligand 1; NPs: nanoparticles; HDL: high-density lipoprotein; IFN-γ: interferon-γ; JAK1/2: Janus kinase 1/2; STAT: signal transducer and activator of transcription; STING: stimulator of interferon genes; CDN: cyclic dinucleotide; PTEN: phosphatase and tensin homolog; PI3K: phosphoinositide 3-kinase; Akt: protein kinase B; mRNA: messenger RNA.

The nanomaterial-based strategies discussed below are not strictly classified according to the specific resistance mechanisms they are intended to overcome, as illustrated in Figure 2. Rather, individual NP formulations may be involved in more than one strategic category, depending on their design characteristics and mechanisms of action.

## NANOMATERIAL-BASED STRATEGIES TO OVERCOME PD-1/PD-L1 PRIMARY RESISTANCE

Recently, nanomaterials have been increasingly engineered to overcome resistance to immunotherapy. Those strategies can not only directly block the immune checkpoint but also remodel the TME to reinvigorate anti-tumor immunity^[54]^. The physicochemical properties of nanomaterials provide unique advantages for precise target delivery, enhancing drug stability, and controlled release^[55]^. These features underscore the potential of nanomaterial-based therapies to overcome the PD-1/PD-L1 primary immune resistance. Based on the currently available nanomaterial-based strategies, we categorized the mechanisms into four major intervention classes: (i) signaling pathway modulation; (ii) checkpoint gene silencing; (iii) directed blockade of the PD-1/PD-L1 axis; and (iv) TME reprogramming.

### Mechanisms of nanomaterial-based strategies

#### Signaling pathway

Signaling pathways play a pivotal role in the tumor intrinsic factors of primary resistance to PD-1/PD-L1 blockade. In particular, oncogenic pathways like PI3K/AKT and Wnt/β-catenin induce immune evasion, while loss of IFN-γ signaling impairs the efficacy of the PD-1/PD-L1 blockade. Thus, focusing on the treatments for PI3K/AKT, Wnt/β-catenin, and IFN-γ pathway will be one of the key mechanisms to overcome the primary immune resistance of PD-1/PD-L1 therapy.

For the IFN-γ pathway, Kateh Shamshiri *et al.* built a non-polyethylene glycolized (HSPC/DSPG/Chol, LIP-F1) liposome and a polyethylene glycolized (HSPC/DSPG/Chol/mPEG2000-DSPE, LIP-F2) liposome encapsulating with IFN-γ. This combination of liposomes and IFN-γ can modulate M2 macrophage and also upregulate the level of IFN-γ in immune cells, which provides an intense anti-tumor immune response^[56]^. Liposome is a type of nanomaterial or nanocarrier that has been discovered by Bangham and colleagues in 1965^[57]^. As people gradually improve the understanding of liposome, liposome-derived technologies are now recognized as one of the cornerstones of bionanotechnology^[58]^. Similar strategies have also been reported by Liu *et al.*, who developed a nebulized liposomal nanoparticle (NP) loaded with cyclic dinucleotide (CDN) (AeroNP-CDN) for delivery to deep lung tumors^[59]^. In addition to nanomaterials like liposomes, other NPs are also essential. Sun *et al.* presented supramolecular NPs called HCJSP to promote the immune response and suppress the PD-L1 expression triggered by IFN-γ signaling^[60]^.

**Figure 3:**
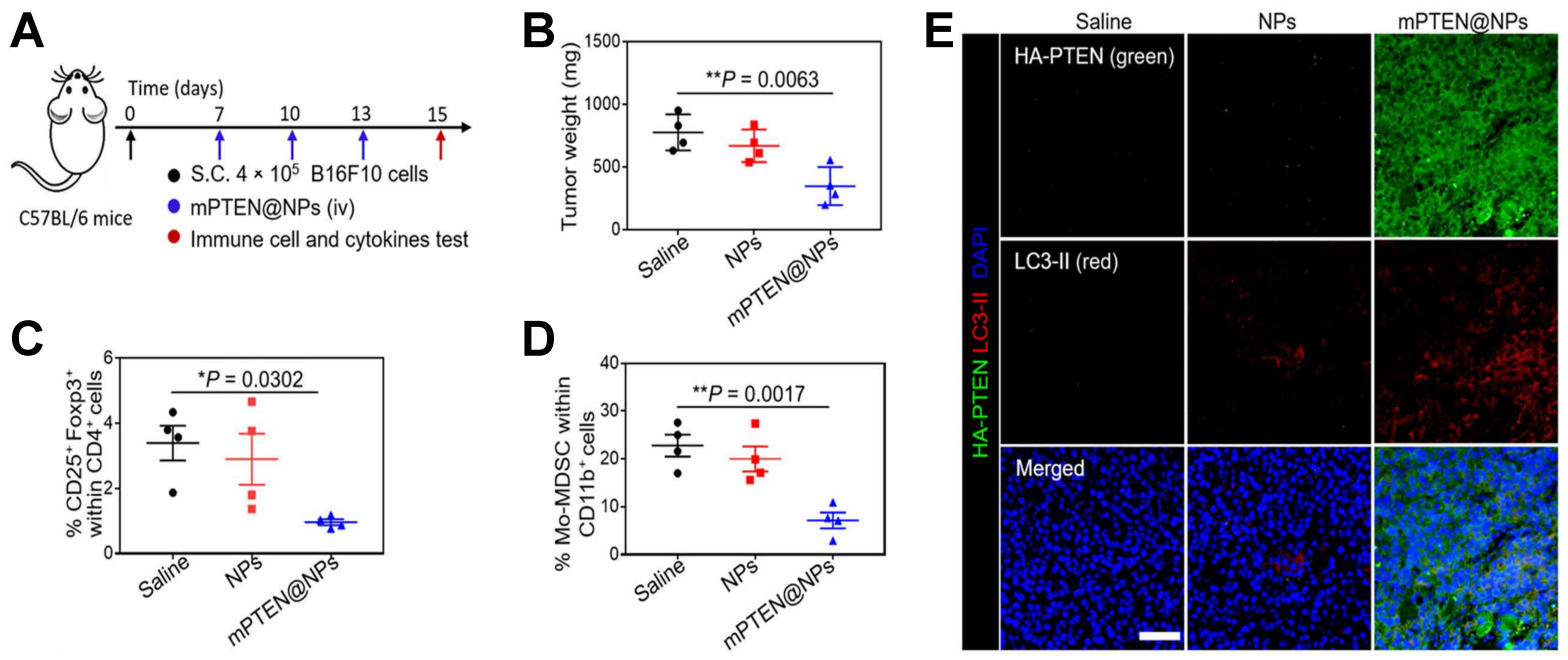
Therapeutic concept and *in vivo* effects of mPTEN@NPs in a B16F10 melanoma model. This figure illustrates mPTEN@NPs as a nanoplatform designed to restore PTEN-related antitumor activity and remodel the immunosuppressive TME, thereby helping to overcome resistance to immunotherapy. The proposed strategy enhances immune activation while reducing suppressive mechanisms associated with tumor progression. Model: B16F10 melanoma. Key readouts: tumor control, lymph node dendritic cell activation, CD8^+^ T-cell infiltration and effector function, reduced Treg and Mo-MDSC populations, cytokine modulation, PTEN and LC3-II expression, and ATP release. (A) Experimental timeline; (B) Tumor weights of B16F10 tumor-bearing mice treated with PTEN@NPs; (C and D) Flow cytometric analysis of the percentages of Foxp3^+^ CD25^+^ CD4^+^ T cells (C) and Mo-MDSCs (D); (E) Immunofluorescence imaging of PTEN (green) and LC3-II (red) in PTEN-mutated B16F10 tumor tissues. Adapted with permission from American Association for the Advancement of Science^[62]^. mPTEN: PTEN mRNA (PTEN: phosphatase and tensin homolog); NPs: nanoparticles; TME: tumor microenvironment; Treg: regulatory T cell; Mo-MDSC: monocytic myeloid-derived suppressor cell; ATP: adenosine triphosphate.

Besides IFN-γ signaling, PI3K/AKT and Wnt/β-catenin are also crucial. Zhang *et al.* demonstrated that an internalizing RGD (iRGD) peptide-modified lipid nanoparticle (LNP). They used it to encapsulate PI3K inhibitor to block the PI3K/AKT signaling pathway, which inhibits tumor-mediated immunosuppression^[61]^. In addition to directly blocking the PI3K/AKT signaling pathway, Lin *et al.* developed a PTEN messenger RNA (mRNA) NP, called mPTEN@NPs, to effectively induce the PTEN expression by the targeted delivering of mRNA to tumor sites, thus restoring the function of lost or mutated PTEN protein^[62]^. The results are shown in Figure 3^[62]^ which suggests that mPTEN@NPs, by restoring PTEN, successfully counteracted the immune resistance caused by PTEN loss. mPTEN@NPs can promote tumor cell apoptosis and inhibit tumor growth [Figure 3B], while also demonstrating the ability to reverse the immunosuppressive TME by reducing the proportions of Tregs [Figure 3C] and monocytic myeloid-derived suppressor cells (Mo-MDSCs) [Figure 3D]. The enhanced immunofluorescence signal of hemagglutinin (HA)-tagged PTEN (HA-PTEN) observed in the mPTEN@NPs-treated group [Figure 3E] indicates that mPTEN@NPs effectively delivered PTEN mRNA to the tumor site, achieving PTEN restoration *in vivo*. Increasing the expression of PTEN is an effective way to counteract the PI3K/AKT pathway and the tumor immunosuppression. Compared with IFN-γ-related nanotechnologies, relatively few nanomaterial-based strategies targeting the PI3K/AKT and Wnt/β-catenin pathways have been reported for overcoming primary resistance to PD-1/PD-L1 blockade. These might be the possible fields that deserve attention in the future.

#### Checkpoint gene silencing

**Figure 4:**
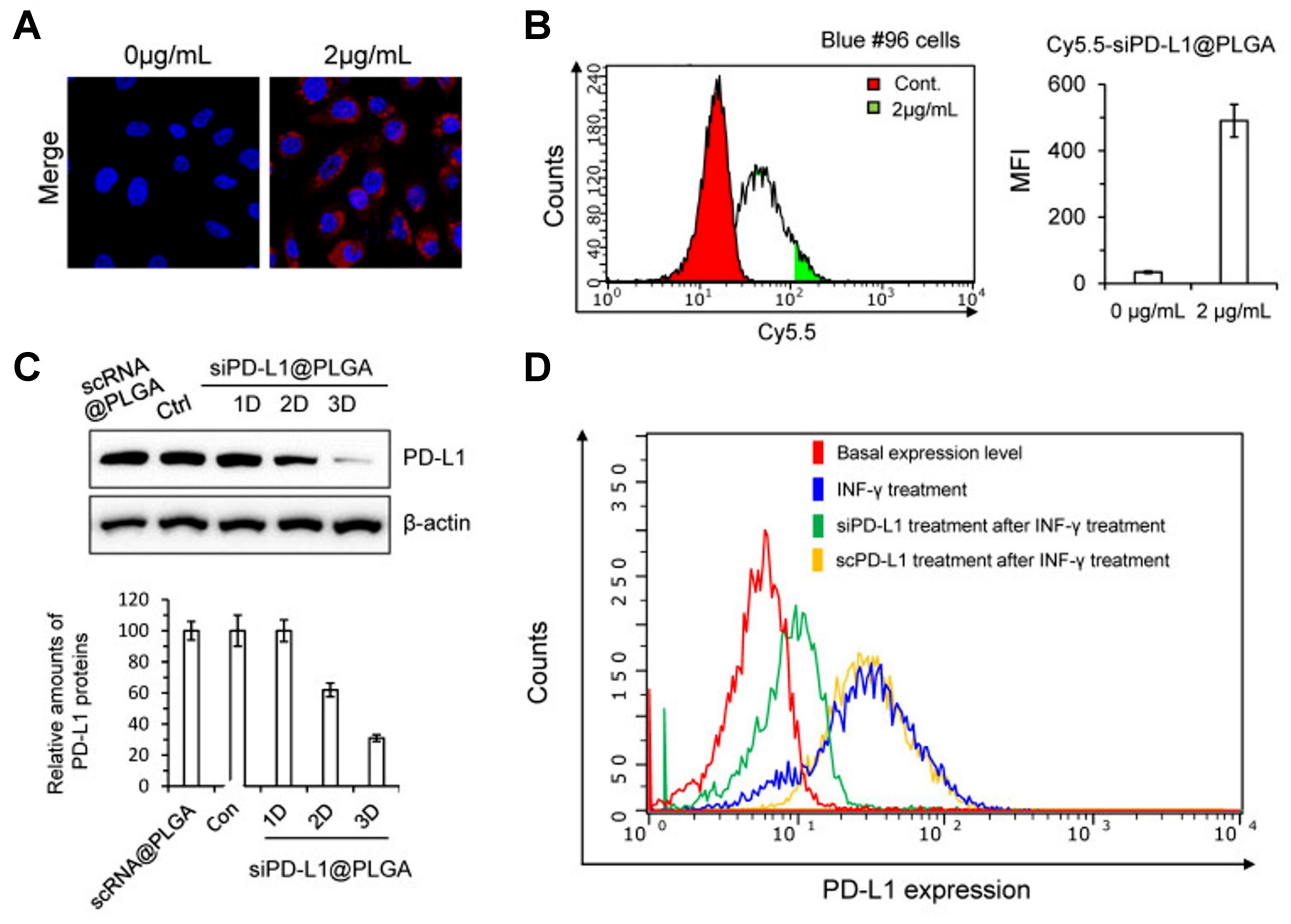
Therapeutic concept and experimental validation of PLGA NPs for siRNA-mediated PD-L1 silencing. This figure illustrates the use of PLGA NPs as a nanoplatform for siRNA-mediated PD-L1 silencing to enhance intracellular delivery and reduce tumor immune evasion. It conceptually shows how NP-enabled gene silencing may mitigate PD-L1-associated resistance mechanisms and improve responsiveness to immunotherapy. Model: pancreatic cancer. Key readouts: NP uptake, intracellular siRNA delivery, PD-L1 knockdown, and inhibition of IFN-γ-induced PD-L1 upregulation. (A) Confocal imaging of Blue #96 cells treated with Cy5.5-scRNA@PLGA NPs, showing robust cellular uptake at a concentration of 2 mg/mL; (B) FACS histogram of Blue #96 cells treated with Cy5.5-scRNA@PLGA, showing substantial cellular uptake of the NPs; (C) Western blot analysis of Blue #96 cells after transfection with siPD-L1@PLGA NPs, showing a marked decrease in PD-L1 expression 2-3 days after treatment; (D) FACS histogram of PD-L1 expression under different treatments, showing that siPD-L1@PLGA reduced IFN-γ-induced PD-L1 expression, whereas scPD-L1@PLGA did not. Reprinted from Multidisciplinary Digital Publishing Institute under a CC BY 4.0 license^[64]^. PLGA: Poly(lactic-co-glycolic acid); NPs: nanoparticles; siRNA: small interfering RNA; PD-L1: programmed death-ligand 1; IFN-γ: interferon-γ; scPD-L1: scrambled siRNA; FACS: fluorescence-activated cell sorting; MFI: mean fluorescence intensity.

Rather than directly modulating signaling pathways, some strategies use siRNA to knock down checkpoint genes. siRNA consists of short double-stranded RNA molecules that can be designed to knock down specific genes^[63]^. Thus, siRNA becomes a potential approach to suppress the expression of PD-L1 proteins in cancer cells. Naked siRNA is rapidly degraded and exhibits poor membrane permeability because of its relatively large molecular size and negative charge. Consequently, nanotechnology can be used in combination with siRNA to improve its therapeutic performance. This is illustrated by the study conducted by Jung *et al.*, in which PD-L1-targeting siRNA NPs, denoted as siPD-L1@PLGA [poly(lactic-co-glycolic acid)], were used for the treatment of pancreatic cancer^[64]^. Jung *et al.* showed that siPD-L1@PLGA effectively silenced PD-L1 in pancreatic cancer cells. Confocal imaging and flow cytometry confirmed efficient NP uptake in Blue #96 cells [Figure 4A and B], while western blotting demonstrated a time-dependent reduction in PD-L1 protein expression after transfection [Figure 4C]. Consistent with these findings, flow cytometric analysis further showed that siPD-L1@PLGA suppressed IFN-γ-induced PD-L1 upregulation, whereas the scrambled control did not [Figure 4D]. Their results indicate that siPD-L1@PLGA effectively suppresses PD-L1 expression in pancreatic cancer cells, thereby enhances antitumor immune responses^[64]^.

A study done by Wu *et al.* used a nanotechnological approach, lipid-coated calcium phosphate NPs (LCN), to deliver siRNA targeting the PD-1/PD-L1 axis, thus preserving the activity of the T cells and reducing the primary resistance of PD-1/PD-L1 immunotherapy. LCN-siRNA PD-1/PD-L1 NPs targeting PD-1 and PD-L1 were administered separately, resulting in enhanced cytotoxic T-cell activity, and improved antitumor efficacy. In this combination silencing, the cytotoxicity was related to the increased release of IFN-γ and tumor necrosis factor-α (TNF-α)^[65]^. Upregulation of these cytokines further contributed to blockade of the PD-1/PD-L1 axis. Similarly, Erel-Akbaba *et al.* developed another NP-based system. They created a tumor-targeting solid lipid nanoparticle (SLN) to carry the siRNA into the brain tumor region against glioblastoma. This combination downregulated the expression of tumor PD-L1 and improved the survival of mice^[66]^.

Apart from siRNA, there is another type of RNA that is able to silence the PD-1/PD-L1 genes, which is named small hairpin RNA of PD-L1 (shPD-L1). Guan *et al.* combined hyaluronidase (HAase), which degrades hyaluronic acid and enhances NP penetration, with an ultrasensitive pH-triggered shPD-L1 nanoplatform, thereby increasing PD-L1 gene silencing and suppressing PD-L1 expression^[67]^.

#### Directed block PD-1/PD-L1

**Figure 5:**
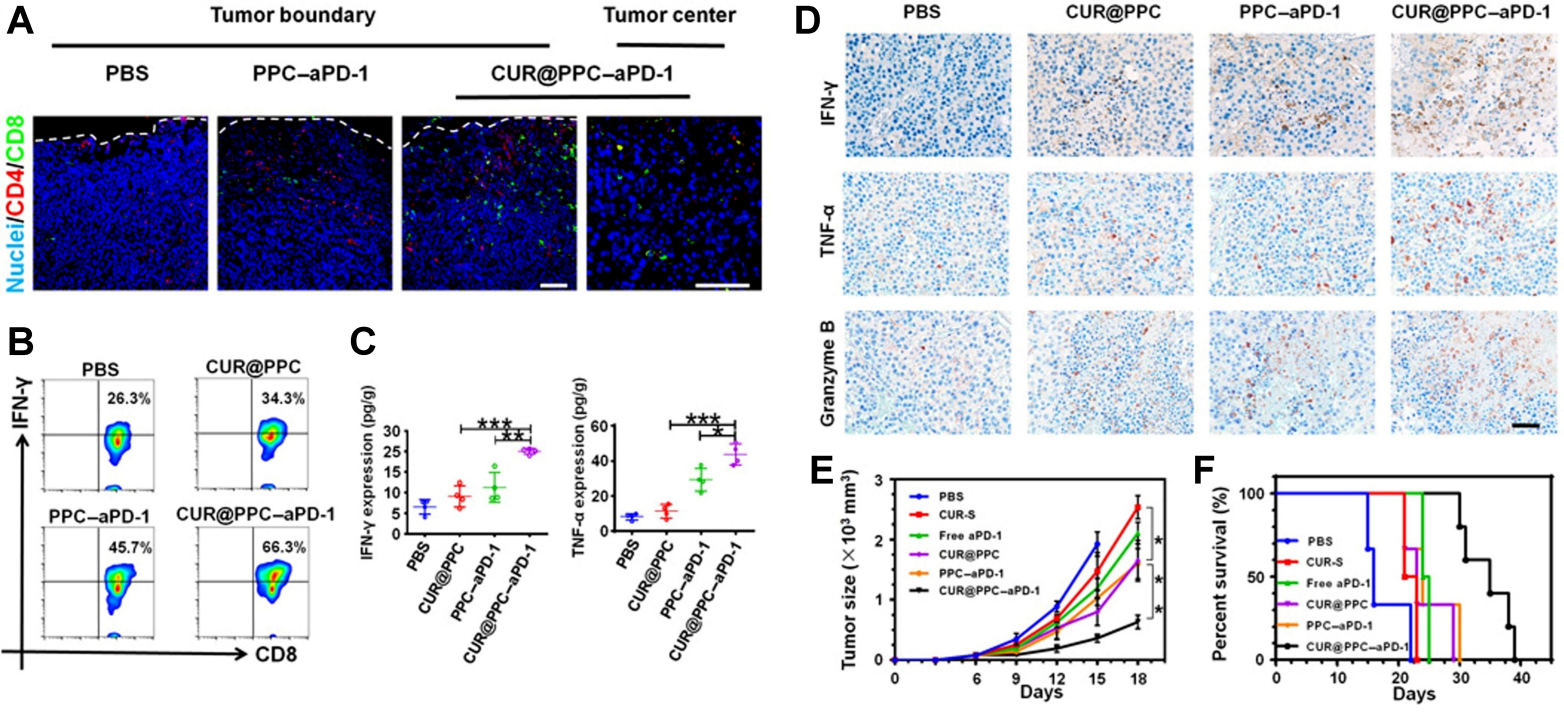
Therapeutic concept and antitumor effects of CUR@PPC-aPD-1 in a B16F10 melanoma model. This figure illustrates CUR@PPC-aPD-1 as an aPD-1-targeted, dual pH-sensitive nanodrug designed to enhance antitumor immunity by combining nanocarrier-mediated drug delivery with immune checkpoint blockade. It conceptually shows how this platform may help overcome PD-1/PD-L1-mediated immunosuppression and improve therapeutic responsiveness within the TME. Model: B16F10 melanoma. Key readouts: PD-1^+^ T-cell tumor infiltration, CD8^+^ IFN-γ^+^ T-cell activation, IFN-γ and TNF-α production, expression of tumoricidal cytokines, tumor growth inhibition, and survival benefit. (A) Immunofluorescence imaging of B16F10 tumors, showing the effect of aPD-1 delivery on tumor infiltration by PD-1^+^ T cells; (B) Flow cytometric analysis of CD8^+^ IFN-γ^+^ T cells, showing that intracellular IFN-γ expression increased in the CUR@PPC and PPC-aPD-1 groups compared with the PBS group, with the highest proportion of IFN-γ-expressing cells observed in the CUR@PPC-aPD-1 group; (C) ELISA analysis of IFN-γ and TNF-α, showing that CUR@PPC-aPD-1 treatment yielded the highest levels of both cytokines; (D) Immunohistochemical staining of tumoricidal cytokines, including IFN-γ, TNF-α, and granzyme B, in B16F10 tumors, showing the strongest upregulation in the CUR@PPC-aPD-1 group; granzyme B is a cytotoxic effector molecule that induces DNA fragmentation; (E and F) Tumor growth and cumulative survival of mice receiving different treatments, showing that CUR@PPC-aPD-1 markedly inhibited tumor growth and significantly improved survival. Data are presented as mean ± SD. ^*^P < 0.05, ^**^P < 0.01, and ^***^P < 0.001. Reprinted with permission from American Association for the Advancement of Science under a CC BY - NC 4.0 license^[70]^. CUR: Curcumin; PPC: PDPA-PEG-CDM; PD-1: programmed cell death protein 1; PD-L1: programmed death-ligand 1; TME: tumor microenvironment; IFN-γ: interferon-γ; TNF-α: tumor necrosis factor-α; PBS: phosphate-buffered saline; ELISA: enzyme-linked immunosorbent assay.

Nanomaterials can be decorated with immune checkpoints or checkpoint-binding ligands to absorb and neutralize PD-1/PD-L1 within tumors. This strategy restores immune activity by antagonizing PD-1/PD-L1 directly at the tumor site. Yin *et al.* designed a novel nanotechnology that combined rapamycin (RAPA)-loaded PLGA and PD-1 overexpressed macrophage membrane to form a NP called PD-1-MM(macrophage-membrane-coated)@PLGA/RAPA. This NP can cross the blood-brain barrier and preferentially accumulate in PD-L1-high tumor regions. PD-1 displayed on the macrophage membrane can bind PD-L1 on tumor cells, thereby functionally blocking PD-1/PD-L1 interactions and inhibiting tumor growth^[68]^. A similar approach was reported by Younis *et al.*, who developed a nanovesicle named IGU-Rh-PD-1, loaded with Iguratimod (IGU) and rhodium (Rh) NPs^[69]^. This nanovesicle can detect the PD-L1 expressed on tumor cells surface and block the PD-1/PD-L1 axis. The IGU in it can inhibit the mTOR signaling pathway, while Rh-NPs induce cancer cell death, thereby reactivating antitumor T-cell responses. In addition, Xiao *et al.* developed a core-shell nanodrug in which the outer layer was coated with aPD-1 and the core encapsulated the nuclear factor kappa B (NF-κB) inhibitor curcumin (CUR). The nanodrug can bind to PD-1^+^ T cells, thus releasing the aPD-1 to block the PD-1 on T cells. Based on Figure 5A^[70]^, CUR@PDPA-PEG-CDM (PPC)-aPD-1 treatment demonstrated superior therapeutic efficacy compared with PPC(the nanocarrier)-aPD-1 treatment, as evidenced by the increased infiltration of CD8^+^ T cells and CD4^+^ T cells. Figure 5B shows a combination effect of CUR and aPD-1 on the activation of CD8^+^ T cell. Comparing with the other groups, the CUR@PPC-aPD-1 treatment provided the best effect to restore the tumor immune microenvironment (TIME) by improving the expression of TNF-α and IFN-γ [Figure 5C]. Immunohistochemical staining further revealed that granzyme B has a stronger signal in the CUR@PPC-aPD-1 group [Figure 5D], consistent with enhanced cytotoxic lymphocyte effector activity upon PD-1/PD-L1 checkpoint inhibition. The pH-sensitive nanomicelle plays a crucial role in ensuring effective delivery of aPD-1 and maximizing its immunotherapeutic efficacy [Figure 5E and F]. CUR was shown to downregulate the expression of cytokines like [C-C motif chemokine ligand 22 (CCL-22), transforming growth factor-beta (TGF-β), and interleukin-10 (IL-10)], which effectively activate the antitumor immunity^[70]^.

#### Modulating the TME

Nanomaterials can be employed to reprogram the TME from a immunosuppressive state to an inflammatory one. This strategy is crucial for reversing extrinsic resistance factors. To modulate the TME, these nanomaterials are first designed to accumulate in tumors through targeted delivery and then to regulate tumor-associated macrophages (TAMs), MDSCs, and other components of the microenvironment to prevent the formation of an immunosuppressive TME^[71]^.

The study by Chen *et al.* developed a combination of a nanovaccine with aPD-1, aOX40, and ibrutinib that enhanced immune-cell infiltration and reduced immunosuppression. To achieve that, the aPD-1 can effectively block the PD-1/PD-L1 pathway; aOX40 can stimulate immune activation and enhanced immune-cell infiltration; and ibrutinib (a MDSCs inhibitor) can counteract MDSCs-mediated immunosuppression. The nanovaccine delivered to the TME will downregulate the level of MDSCs and block the PD-1/PD-L1 pathway, thus promoting the immune response and avoiding the immune resistance of PD-1/PD-L1^[72]^. In order to mediate the immunosuppressive TME, there are other studies that developed effective strategies like lipid-encapsulated calcium phosphate NPs loaded with gemcitabine to exhaust MDSCs^[73]^, and mesoporous silica NPs loaded with all-trans retinoic acid and doxorubicin, coated with IL-2 and subsequently modified with dipalmitoyl phosphatidylcholine cholesterol and DSPE-PEG 2000 to reduce the MDSCs population^[74]^.

In addition to MDSCs, Wang *et al.* designed an aPD-L1/indocyanine green (ICG)-based TIME-sensitivenanoparticle (S-aPD-L1/ICG@NP) to activate T cells by blocking the overexpressed PD-L1 on the surface of tumor cells within the TIME. Other than blocking PD-1/PD-L1, the tumor-infiltrating CD8^+^ T cell ratio and secretion of IFN-γ and TNF-α are also increased thereby enhancing antitumor immune response^[75]^.

The summary list of representative nano-platforms are included in the Table 1.

**Table 1:** Summary of representative nano-platforms to overcome PD-1/PD-L1 resistance

**Platform**	**Payload**	**Target/pathway**	**Setting (primary *vs.* acquired)**	**Evidence level (*in vitro*/*in vivo*/phase I/phase II)**	**Cancer model**	**Endpoints**
Nebulized liposomal NP (AeroNP-CDN)	STING agonist (c-di-GMP)	STING pathway; IFN genes	Acquired	*In vivo*	Lung tumor model	↑ IFN-stimulated genes in TAMs; ↓ IFN-γ-driven PD-L1 upregulation in tumor cells^[59]^
HCJSP supramolecular NP	Peptide/polymeric complex	IFN-γ; PD-L1 axis	Acquired	*In vivo*	Pancreatic cancer	↑ DC activation; ↓ tumor PD-L1 expression; enhanced T cell killing^[60]^
iRGD peptide-modified lipid NP	PI3K inhibitor (small molecule)	PI3K/AKT pathway	Primary	*In vivo*	4T1 breast cancer	↓ PI3K/AKT signaling; reduced tumor immunosuppression; ↑ T cell activity^[61]^
Polymeric NP	PTEN mRNA	Restores PTEN; antagonizes PI3K/AKT	Primary	*In vivo*	PTEN-null melanoma; PTEN-null prostate	↑ PTEN expression; ↑ cell death (autophagy); ↑ CD8^+^ T cells; cytokines; ↑ Tregs/MDSCs^[62]^
PLGA NP	siPD-L1 (siRNA)	PD-L1 gene silencing	Primary	*In vivo*	Pancreatic cancer	↓ Tumor PD-L1; ↑ CD8^+^ T cells; tumor growth inhibition^[64]^
LCN	siPD-1 and siPD-L1 (siRNAs)	PD-1/PD-L1 blockade	Primary	*In vivo*	breast cancer	↑ CD8^+^ T cell cytotoxicity; ↑ IFN-γ, TNF-α; improved tumor control^[65]^
Tumor-targeting SLN	siPD-L1 (siRNA)	PD-L1 silencing	Primary	*In vivo*	Glioblastoma	↓ Tumor PD-L1; ↑ survival of tumor-bearing mice^[66]^
HAase/pH-sensitive NP	shPD-L1 (shRNA)	PD-L1 gene silencing	Primary	*In vivo*	Malignant melanoma	↑ Tumor penetration; ↑ PD-L1 knockdown; enhanced immune response^[67]^
Macrophage-membrane-coated PLGA NP (PD-1-MM@PLGA)	PD-1 on membrane + RAPA	PD-1/PD-L1 blockade; mTOR inhibition	Primary	*In vivo*	Glioblastoma	BBB penetration; ↓ tumor PD-L1; tumor growth inhibition^[68]^
PD-1/LAG-3-decorated nanovesicle (IGU-Rh-PD-1)	IGU + Rh NPs	PD-1/PD-L1 blockade; mTOR pathway	Primary	*In vivo*	Lung cancer	↓ Tumor PD-L1; ↑ T cell activation; tumor suppression^[69]^
Dual pH-sensitive nanodrug (CUR@PPC-aPD-1)	CUR + anti-PD-1 Ab	PD-1 blockade; NF-κB inhibition	Primary	*In vivo*	B16F10 melanoma	↑ Tumor-infiltrating CD8^+^/CD4^+^; ↑ IFN-γ/TNF-α; ↓ tumor growth; ↑ survival^[70]^
Combination of nanovaccine with aPD-1, aOX40, and ibrutinib	aPD-1 + aOX40 + ibrutinib	MDSC inhibition	Primary	*In vivo*	Breast cancer	↓ MDSCs; block the PD-1/PD-L1 pathway^[72]^
Gemcitabine-CaP NP (lipid-coated)	Gemcitabine	MDSC depletion	Acquired	*In vivo*	4T1 mammary carcinoma	↓ MDSCs; ↑ response to PD-L1 blockade^[73]^
aPD-L1/ICG TIME-sensitive NP	aPD-L1 antibody + ICG	PD-L1 blockade	Primary	*In vivo*	Melanoma	↑ Tumor-infiltrating CD8^+^ T cells; ↑ IFN-γ/TNF-α; enhanced tumor regression^[75]^

Each row details the nanocarrier type, therapeutic payload(s), intended target/pathway, *in vivo* cancer model, and main outcomes. PD-1: Programmed cell death protein 1; PD-L1: programmed death-ligand 1; NP: nanoparticle; CDN: cyclic dinucleotide; STING: stimulator of interferon genes; GMP: good manufacturing practice; IFN: interferon; TAMs: tumor-associated macrophages; DC: dendritic cell; iRGD: internalizing RGD; PI3K: phosphoinositide 3-kinase; AKT: protein kinase B; PTEN: phosphatase and tensin homolog; mRNA: messenger RNA; MDSCs: myeloid-derived suppressor cells; PLGA: poly(lactic-co-glycolic acid); siRNA: small interfering RNA; LCN: lipid-coated calcium phosphate NPs; TNF-α: tumor necrosis factor-α; SLN: solid lipid nanoparticle; HAase: hyaluronidase; shPD-L1: small hairpin RNA of PD-L1; RAPA: rapamycin; mTOR: mechanistic target of rapamycin; BBB: blood-brain barrier; LAG-3: lymphocyte-activation gene 3; IGU: Iguratimod; Rh: rhodium; CUR: curcumin; PPC: PDPA-PEG-CDM; NF-κB: nuclear factor kappa B; ICG: indocyanine green; TIME: tumor immune microenvironment.

## CHALLENGES AND CLINICAL TRANSLATION CONSIDERATIONS FOR NANOMATERIAL-ENABLED PD-1/PD-L1 IMMUNOTHERAPY

### Why translation remains difficult

Nanomaterial-based approaches, such as NPs carrying checkpoint-blocking biologics, pathway inhibitors, or nucleic acids to sensitize tumors to PD-1/PD-L1 therapy, are conceptually promising. However, recent translational analyses have shown that several fundamental barriers continue to prevent or delay clinical success. These barriers include limited exposure at the target tissue/cell, incomplete understanding of how physicochemical attributes influence *in vivo* performance, poor reproducibility of preclinical outcomes in clinical trials, biocompatibility concerns, and downstream bottlenecks such as industrial scale-up, good manufacturing practice (GMP)-compliant manufacturing and regulatory navigation^[76]^. In parallel, oncology-focused delivery reviews highlight that even highly mature nucleic acid-based nanoplatforms (e.g., mRNA-LNPs) face interconnected physiological, technological, and manufacturing challenges before they can reliably deliver clinical benefit, especially when positioned to complement or improve established immunotherapies^[77]^.

#### Delivery heterogeneity and the “EPR gap” between models and patients

A central translational challenge is that many nano-immunotherapy concepts still depend on passive tumor accumulation through the enhanced permeability and retention (EPR) effect. A mechanistic and clinically oriented review in Journal of Controlled Release concludes that the EPR effect is highly variable and thus unreliable because of TME complexity, and stresses that understanding differences between animal and human tumors is essential for translation^[78]^.

Clinical inconsistency is also driven by methodological limitations. A recent study notes that, despite the widespread use of the EPR concept, clinical outcomes remain inconsistent, in part due to limited mechanistic understanding and tools to quantify delivery-relevant phenomena *in vivo*^[79]^. This consideration is particularly important for PD-1/PD-L1-targeted combination nanotherapies, because delivery to the tumor region does not necessarily ensure penetration into the tumor parenchyma or adequate exposure of the immune and tumor cell subsets that the response to PD-1/PD-L1 blockade^[79]^.

#### Immunogenicity and immune system interactions that can counteract benefit

For PD-1/PD-L1 nano-immunotherapy, delivery systems are not neutral containers. They can reshape immune responses in ways that alter efficacy, safety, and the feasibility of repeated dosing. In PEGylated systems, some individuals have pre-existing anti-PEG (polyethylene glycol) antibodies and that PEG-modified compounds can induce additional anti-PEG antibodies. These antibodies can adversely affect drug efficacy and safety, including through accelerated blood clearance^[80]^.

Mechanistically, a study shows that anti-PEG antibodies can trigger complement activation on PEGylated lipid-based NPs (including mRNA-LNPs). This process may compromise NP integrity and lead to premature drug release or increased exposure of the cargo to serum proteins. The same study also reported correlations between pre-existing anti-PEG IgM levels and complement activation in human donor plasma^[81]^.

Beyond PEG, infusion-related hypersensitivity is increasingly recognized as a concern for LNPs. A review on hypersensitivity to mRNA-LNP vaccines argues that rare anaphylaxis-like events resemble infusion reactions previously observed with nanomedicines. It also emphasized the need for reliable predictive tools and safer strategies for repeated administration, which are directly relevant to the design of nanocarriers intended for repeated dosing alongside PD-1/PD-L1 therapy^[82]^.

#### Safety, biodistribution, and toxicity risks in immune-oncology settings

For translational development, toxicity is not limited to the active payload. Nanomaterial composition, production variables, route of administration, and tissue distribution patterns can all contribute to safety liabilities. A recent article emphasizes that avoiding unacceptable toxicity with mRNA drugs and vaccines remains challenging. It specifically discussed how cell tropism and tissue distribution of mRNA and LNPs can lead to toxicity and reactogenicity, while also highlighted the limitations of current models for de-risking off-target toxicity^[83]^.

From a broader nanomedicine translation standpoint, the DELIVER framework in Caputo’s article explicitly lists biocompatibility concerns and limited exposure at the target tissue and cell as critical barriers to clinical success^[76]^. These issues are particularly relevant to PD-1/PD-L1 strategies, because the therapeutic goal is to improve antitumor immunity without provoking unacceptable systemic immune activation. Accordingly, biodistribution and immune compatibility should be considered core design constraints rather than post hoc concerns^[83]^.

### Bottlenecks in PD-1/PD-L1 clinical development

In the current PD-1/PD-L1 landscape, in which clinically effective monoclonal antibody therapies are already well established, nanoformulations must demonstrate clear incremental value. This may include improving intratumoral exposure in clinically relevant settings, enhancing the therapeutic index, or providing measurable clinical benefit in the setting of resistance. At the same time, transparent and systematic safety monitoring is essential to address risks jointly driven by both the payload and the carrier. Translational frameworks further identify limited reproducibility in the pathway from preclinical studies to clinical outcomes as a common mode of failure, underscoring the need for methodological improvements and greater standardization^[76]^.

## CLINICAL-STAGE NANO-IMMUNOTHERAPY FORMULATIONS EVALUATED WITH PD-1/PD-L1 BLOCKADE

Based on recent clinical studies, nanotechnology-enabled immunotherapy strategies combined with PD-1/PD-L1 blockade can be broadly categorized into two major classes: (i) systemically administered cancer vaccines delivered via NPs; and (ii) intratumorally administered particulate formulations intended to enhance and potentially restore responsiveness to PD-1-based therapy.

The personalized neoantigen mRNA is delivered using LNPs, and is used in combination with the anti-PD-1 monoclonal antibody pembrolizumab for the treatment of PD-1 inhibitor-resistant melanoma. In the KEYNOTE-942 clinical trial, an open-label, randomized phase IIb study involving patients with cutaneous melanoma at high risk of recurrence after complete surgical resection, researchers compared adjuvant treatment with personalized neoantigen mRNA therapy mRNA-4157 (V940) plus pembrolizumab *vs.* pembrolizumab alone. The mRNA-4157 (V940) vaccine was encapsulated in LNPs to enable efficient delivery. Combination therapy prolonged relapse-free survival (RFS), with 18-month RFS rates of 79% and 62%, respectively, while maintaining a manageable safety profile^[84]^.

Another example is the combination therapy of virus-like particle (VLP)-based TLR9 ligand vidutolimod (CMP-001) and anti-PD-1 monoclonal antibody pembrolizumab. In the dose-escalation phase of a phase Ib clinical trial, patients with advanced melanoma whose disease had progressed after anti-PD-1 immunotherapy received intratumoral vidutolimod, a CpG-A TLR9 agonist formulated using VLPs, together with intravenous pembrolizumab. The treatment showed an acceptable safety profile, and 25% of patients achieved durable clinical responses. Notably, tumor volume was reduced not only in injected lesions but also in noninjected lesions, including visceral metastases^[85]^.

In advanced melanoma, intratumoral administration of the nanocomplex BO-112, a poly I:C formulation complexed with polyethyleneimine, has also been evaluated in combination with intravenous pembrolizumab in the phase II SPOTLIGHT-203 trial in patients with acquired resistance to PD-1 inhibitors. In the intention-to-treat population, the independently assessed objective response rate was 25% , including 10% complete response and 15% partial responses. The median time to progression was 3.7 months and an overall survival probability of 54% at 24 months. Safety was acceptable, with no treatment-related fatal adverse events reported^[86]^. Although this trial addresses acquired resistance to PD-1 blockade rather than primary resistance, it illustrates how nano-enabled immune stimulation strategies are being explored clinically in PD-1-refractory populations.

Based on the recent literature, clinical-stage nano-strategies directly designed to counter tumor-intrinsic resistance pathways, such as Wnt/β-catenin, PTEN/PI3K-AKT, or defects in IFN signaling, remain scarce. Most pathway-matched nanosystems, including PTEN mRNA NPs, PD-1 membrane decoys, and PD-L1 siRNA NPs, are still at the preclinical stage. This gap highlights the need for biomarker-driven trial designs and scalable, regulatory-ready formulations.

## CONCLUSION

Primary (intrinsic) resistance to PD-1/PD-L1 blockade remains a major barrier to durable responses across cancer types. Mechanistically, primary resistance can arise from tumor intrinsic alterations (e.g., defects in IFN signaling, PTEN loss with PI3K/AKT activation, or Wnt/β-catenin-associated immune exclusion) and tumor-extrinsic immunosuppressive programs (e.g., MDSC- and macrophage-mediated suppression) that converge on impaired T-cell priming, trafficking, and effector function.

Nanomaterial-enabled strategies provide a flexible toolkit to address these determinants by (i) modulating resistance-associated signaling pathways; (ii) enabling localized or multivalent interception of the PD-1/PD-L1 axis; (iii) silencing checkpoint genes using nucleic-acid delivery platforms; and (iv) reprogramming the TME to favor productive antitumor immunity. Nevertheless, most mechanistically matched nano-interventions remain preclinical, and translation will require biomarker-guided patient stratification, scalable GMP-compliant manufacturing, rigorous safety evaluation, and reproducible efficacy across clinically relevant models.

## DECLARATIONS

### AI and AI-assisted tools statement

During the preparation of this manuscript, the AI tool ChatGPT (version 5.4, released 2026-03-05) was used solely for language editing. The tool did not influence the study design, data collection, analysis, interpretation, or the scientific content of the work. All authors take full responsibility for the accuracy, integrity, and final content of the manuscript.

### Copyright

© The Author(s) 2026.

## References

[1] Yin Q, Wu L, Han L (2023). Immune-related adverse events of immune checkpoint inhibitors: a review. Front Immunol.

[2] Shiravand Y, Khodadadi F, Kashani SMA (2022). Immune checkpoint inhibitors in cancer therapy. Curr Oncol.

[3] Johnson DB, Nebhan CA, Moslehi JJ, Balko JM (2022). Immune-checkpoint inhibitors: long-term implications of toxicity. Nat Rev Clin Oncol.

[4] Mandal K, Barik GK, Santra MK (2025). Overcoming resistance to anti-PD-L1 immunotherapy: mechanisms, combination strategies, and future directions. Mol Cancer.

[5] Zhang D, Zhao J, Zhang Y, Jiang H, Liu D (2024). Revisiting immune checkpoint inhibitors: new strategies to enhance efficacy and reduce toxicity. Front Immunol.

[6] Alsaafeen BH, Ali BR, Elkord E (2025). Resistance mechanisms to immune checkpoint inhibitors: updated insights. Mol Cancer.

[7] Dang BTN, Kwon TK, Lee S, Jeong JH, Yook S (2024). Nanoparticle-based immunoengineering strategies for enhancing cancer immunotherapy. J Control Release.

[8] Lian S, Yang W, Zeng Y, Tang R, Wang K (2026). Targeted nano-drug delivery systems for tumor immunotherapy. J Pharm Anal.

[9] Ishida Y, Agata Y, Shibahara K, Honjo T (1992). Induced expression of PD-1, a novel member of the immunoglobulin gene superfamily, upon programmed cell death. EMBO J.

[10] Lin X, Kang K, Chen P (2024). Regulatory mechanisms of PD-1/PD-L1 in cancers. Mol Cancer.

[11] Freeman GJ, Long AJ, Iwai Y (2000). Engagement of the PD-1 immunoinhibitory receptor by a novel B7 family member leads to negative regulation of lymphocyte activation. J Exp Med.

[12] Jiang Y, Chen M, Nie H, Yuan Y (2019). PD-1 and PD-L1 in cancer immunotherapy: clinical implications and future considerations. Hum Vaccin Immunother.

[13] Kythreotou A, Siddique A, Mauri FA, Bower M, Pinato DJ (2018). PD-L1. J Clin Pathol.

[14] Munari E, Mariotti FR, Quatrini L (2021). PD-1/PD-L1 in cancer: pathophysiological, diagnostic and therapeutic aspects. Int J Mol Sci.

[15] Sharpe AH, Wherry EJ, Ahmed R, Freeman GJ (2007). The function of programmed cell death 1 and its ligands in regulating autoimmunity and infection. Nat Immunol.

[16] Huang D, Wen W, Liu X, Li Y, Zhang JZH (2019). Computational analysis of hot spots and binding mechanism in the PD-1/PD-L1 interaction. RSC Adv.

[17] Paillon N, Mouro V, Dogniaux S (2023). PD-1 inhibits T cell actin remodeling at the immunological synapse independently of its signaling motifs. Sci Signal.

[18] Sharma P, Hu-Lieskovan S, Wargo JA, Ribas A (2017). Primary, adaptive, and acquired resistance to cancer immunotherapy. Cell.

[19] Doroshow DB, Bhalla S, Beasley MB (2021). PD-L1 as a biomarker of response to immune-checkpoint inhibitors. Nat Rev Clin Oncol.

[20] Bai R, Chen N, Li L (2020). Mechanisms of cancer resistance to immunotherapy. Front Oncol.

[21] Monu NR, Frey AB (2012). Myeloid-derived suppressor cells and anti-tumor T cells: a complex relationship. Immunol Invest.

[22] Ruan WS, Feng MX, Xu J (2020). Early activation of myeloid-derived suppressor cells participate in sepsis-induced immune suppression via PD-L1/PD-1 axis. Front Immunol.

[23] Lu C, Redd PS, Lee JR, Savage N, Liu K (2016). The expression profiles and regulation of PD-L1 in tumor-induced myeloid-derived suppressor cells. Oncoimmunology.

[24] Ibrahim A, Mohamady Farouk Abdalsalam N, Liang Z (2025). MDSC checkpoint blockade therapy: a new breakthrough point overcoming immunosuppression in cancer immunotherapy. Cancer Gene Ther.

[25] Zhong C, Niu Y, Liu W (2022). S100A9 derived from chemoembolization-induced hypoxia governs mitochondrial function in hepatocellular carcinoma progression. Adv Sci.

[26] Ikeda H, Old LJ, Schreiber RD (2002). The roles of IFN gamma in protection against tumor development and cancer immunoediting. Cytokine Growth Factor Rev.

[27] Rieth J, Subramanian S (2018). Mechanisms of intrinsic tumor resistance to immunotherapy. Int J Mol Sci.

[28] Gao J, Shi LZ, Zhao H (2016). Loss of IFN-γ pathway genes in tumor cells as a mechanism of resistance to anti-CTLA-4 therapy. Cell.

[29] Gato-Cañas M, Zuazo M, Arasanz H (2017). PDL1 signals through conserved sequence motifs to overcome interferon-mediated cytotoxicity. Cell Rep.

[30] Doi T, Ishikawa T, Okayama T (2017). The JAK/STAT pathway is involved in the upregulation of PD-L1 expression in pancreatic cancer cell lines. Oncol Rep.

[31] Li P, Huang T, Zou Q (2019). FGFR2 promotes expression of PD-L1 in colorectal cancer via the JAK/STAT3 signaling pathway. J Immunol.

[32] Darnell JE Jr, Kerr IM, Stark GR (1994). Jak-STAT pathways and transcriptional activation in response to IFNs and other extracellular signaling proteins. Science.

[33] Shin DS, Zaretsky JM, Escuin-Ordinas H (2017). Primary resistance to PD-1 blockade mediated by JAK1/2 mutations. Cancer Discov.

[34] Manguso RT, Pope HW, Zimmer MD (2017). *In vivo* CRISPR screening identifies Ptpn2 as a cancer immunotherapy target. Nature.

[35] Kalbasi A, Ribas A (2020). Tumour-intrinsic resistance to immune checkpoint blockade. Nat Rev Immunol.

[36] Xue C, Chu Q, Shi Q, Zeng Y, Lu J, Li L (2025). Wnt signaling pathways in biology and disease: mechanisms and therapeutic advances. Signal Transduct Target Ther.

[37] Spranger S, Bao R, Gajewski TF (2015). Melanoma-intrinsic β-catenin signalling prevents anti-tumour immunity. Nature.

[38] Castagnoli L, Cancila V, Cordoba-Romero SL (2019). WNT signaling modulates PD-L1 expression in the stem cell compartment of triple-negative breast cancer. Oncogene.

[39] Huang T, Li F, Cheng X (2021). Wnt inhibition sensitizes PD-L1 blockade therapy by overcoming bone marrow-derived myofibroblasts-mediated immune resistance in tumors. Front Immunol.

[40] Wang H, Luo K, Zhan Y, Peng S, Fan S, Wang W (2023). Role of β-catenin in PD-L1 expression of nasopharyngeal carcinoma. Heliyon.

[41] Sayaman RW, Saad M, Thorsson V (2021). Germline genetic contribution to the immune landscape of cancer. Immunity.

[42] Haddadi N, Lin Y, Travis G, Simpson AM, Nassif NT, McGowan EM (2018). PTEN/PTENP1: 'Regulating the regulator of RTK-dependent PI3K/Akt signalling', new targets for cancer therapy. Mol Cancer.

[43] Chen J, Jiang CC, Jin L, Zhang XD (2016). Regulation of PD-L1: a novel role of pro-survival signalling in cancer. Ann Oncol.

[44] Bergholz JS, Wang Q, Wang Q (2023). PI3Kβ controls immune evasion in PTEN-deficient breast tumours. Nature.

[45] Matsushita H, Vesely MD, Koboldt DC (2012). Cancer exome analysis reveals a T-cell-dependent mechanism of cancer immunoediting. Nature.

[46] Mittendorf EA, Philips AV, Meric-Bernstam F (2014). PD-L1 expression in triple-negative breast cancer. Cancer Immunol Res.

[47] Cui JW, Li Y, Yang Y (2024). Tumor immunotherapy resistance: revealing the mechanism of PD-1 / PD-L1-mediated tumor immune escape. Biomed Pharmacother.

[48] Crane CA, Panner A, Murray JC (2009). PI(3) kinase is associated with a mechanism of immunoresistance in breast and prostate cancer. Oncogene.

[49] Peng W, Chen JQ, Liu C (2016). Loss of PTEN promotes resistance to T cell-mediated immunotherapy. Cancer Discov.

[50] Parsa AT, Waldron JS, Panner A (2007). Loss of tumor suppressor PTEN function increases B7-H1 expression and immunoresistance in glioma. Nat Med.

[51] Zhao R, Song Y, Wang Y (2019). PD-1/PD-L1 blockade rescue exhausted CD8+ T cells in gastrointestinal stromal tumours via the PI3K/Akt/mTOR signalling pathway. Cell Prolif.

[52] Amornsupak K, Thongchot S, Thinyakul C (2022). HMGB1 mediates invasion and PD-L1 expression through RAGE-PI3K/AKT signaling pathway in MDA-MB-231 breast cancer cells. BMC Cancer.

[53] Guo F, Kong W, Li D (2024). M2-type tumor-associated macrophages upregulated PD-L1 expression in cervical cancer via the PI3K/AKT pathway. Eur J Med Res.

[54] Xu Y, Xiong J, Sun X, Gao H (2022). Targeted nanomedicines remodeling immunosuppressive tumor microenvironment for enhanced cancer immunotherapy. Acta Pharm Sin B.

[55] Tian M, Liu X, Pei H (2024). Nanomaterial-based cancer immunotherapy: enhancing treatment strategies. Front Chem.

[56] (2021). Kateh Shamshiri M, Jaafari MR, Badiee A. Preparation of liposomes containing IFN-gamma and their potentials in cancer immunotherapy: *in vitro* and* in vivo* studies in a colon cancer mouse model. Life Sci.

[57] Jesorka A, Orwar O (2008). Liposomes: technologies and analytical applications. Annu Rev Anal Chem.

[58] Leduc PR, Wong MS, Ferreira PM (2007). Towards an in vivo biologically inspired nanofactory. Nat Nanotechnol.

[59] Liu Y, Crowe WN, Wang L, Petty WJ, Habib AA, Zhao D (2023). Aerosolized immunotherapeutic nanoparticle inhalation potentiates PD-L1 blockade for locally advanced lung cancer. Nano Res.

[60] Sun F, Zhu Q, Li T (2021). Regulating glucose metabolism with prodrug nanoparticles for promoting photoimmunotherapy of pancreatic cancer. Adv Sci.

[61] Zhang F, Stephan SB, Ene CI, Smith TT, Holland EC, Stephan MT (2018). Nanoparticles that reshape the tumor milieu create a therapeutic window for effective T-cell therapy in solid malignancies. Cancer Res.

[62] Lin YX, Wang Y, Ding J (2021). Reactivation of the tumor suppressor PTEN by mRNA nanoparticles enhances antitumor immunity in preclinical models. Sci Transl Med.

[63] Yu X, Fang C, Zhang K, Su C (2022). Recent advances in nanoparticles-based platforms targeting the PD-1/PD-L1 pathway for cancer treatment. Pharmaceutics.

[64] Jung JY, Ryu HJ, Lee SH (2021). siRNA nanoparticle targeting PD-L1 activates tumor immunity and abrogates pancreatic cancer growth in humanized preclinical model. Cells.

[65] Wu Y, Gu W, Li J, Chen C, Xu ZP (2019). Silencing PD-1 and PD-L1 with nanoparticle-delivered small interfering RNA increases cytotoxicity of tumor-infiltrating lymphocytes. Nanomedicine.

[66] Erel-Akbaba G, Carvalho LA, Tian T (2019). Radiation-induced targeted nanoparticle-based gene delivery for brain tumor therapy. ACS Nano.

[67] Guan X, Lin L, Chen J (2019). Efficient PD-L1 gene silence promoted by hyaluronidase for cancer immunotherapy. J Control Release.

[68] Yin T, Fan Q, Hu F (2022). Engineered macrophage-membrane-coated nanoparticles with enhanced PD-1 expression induce immunomodulation for a synergistic and targeted antiglioblastoma activity. Nano Lett.

[69] Younis M, Wu Y, Fang Q, Shan H, Huang X (2023). Synergistic therapeutic antitumor effect of PD-1 blockade cellular vesicles in combination with Iguratimod and Rhodium nanoparticles. J Colloid Interface Sci.

[70] Xiao Z, Su Z, Han S, Huang J, Lin L, Shuai X (2020). Dual pH-sensitive nanodrug blocks PD-1 immune checkpoint and uses T cells to deliver NF-κB inhibitor for antitumor immunotherapy. Sci Adv.

[71] Shi Y, Lammers T (2019). Combining nanomedicine and immunotherapy. Acc Chem Res.

[72] Chen IX, Chauhan VP, Posada J (2019). Blocking CXCR4 alleviates desmoplasia, increases T-lymphocyte infiltration, and improves immunotherapy in metastatic breast cancer. Proc Natl Acad Sci U S A.

[73] Le HK, Graham L, Cha E, Morales JK, Manjili MH, Bear HD (2009). Gemcitabine directly inhibits myeloid derived suppressor cells in BALB/c mice bearing 4T1 mammary carcinoma and augments expansion of T cells from tumor-bearing mice. Int Immunopharmacol.

[74] Kong M, Tang J, Qiao Q (2017). Biodegradable hollow mesoporous silica nanoparticles for regulating tumor microenvironment and enhancing antitumor efficiency. Theranostics.

[75] Wang D, Wang T, Yu H (2019). Engineering nanoparticles to locally activate T cells in the tumor microenvironment. Sci Immunol.

[76] Joyce P, Allen CJ, Alonso MJ (2024). A translational framework to DELIVER nanomedicines to the clinic. Nat Nanotechnol.

[77] Kon E, Ad-El N, Hazan-Halevy I, Stotsky-Oterin L, Peer D (2023). Targeting cancer with mRNA-lipid nanoparticles: key considerations and future prospects. Nat Rev Clin Oncol.

[78] Ikeda-Imafuku M, Wang LL, Rodrigues D, Shaha S, Zhao Z, Mitragotri S (2022). Strategies to improve the EPR effect: a mechanistic perspective and clinical translation. J Control Release.

[79] Cooley MB, Wegierak D, Perera R (2024). Assessing therapeutic nanoparticle accumulation in tumors using nanobubble-based contrast-enhanced ultrasound imaging. ACS Nano.

[80] Chen BM, Cheng TL, Roffler SR (2021). Polyethylene glycol immunogenicity: theoretical, clinical, and practical aspects of anti-polyethylene glycol antibodies. ACS Nano.

[81] Estapé Senti M, de Jongh CA, Dijkxhoorn K (2022). Anti-PEG antibodies compromise the integrity of PEGylated lipid-based nanoparticles via complement. J Control Release.

[82] Szebeni J, Storm G, Ljubimova JY (2022). Applying lessons learned from nanomedicines to understand rare hypersensitivity reactions to mRNA-based SARS-CoV-2 vaccines. Nat Nanotechnol.

[83] Bitounis D, Jacquinet E, Rogers MA, Amiji MM (2024). Strategies to reduce the risks of mRNA drug and vaccine toxicity. Nat Rev Drug Discov.

[84] Weber JS, Carlino MS, Khattak A (2024). Individualised neoantigen therapy mRNA-4157 (V940) plus pembrolizumab versus pembrolizumab monotherapy in resected melanoma (KEYNOTE-942): a randomised, phase 2b study. Lancet.

[85] Ribas A, Medina T, Kirkwood JM (2021). Overcoming PD-1 blockade resistance with CpG-A Toll-like receptor 9 agonist vidutolimod in patients with metastatic melanoma. Cancer Discov.

[86] Márquez-Rodas I, Dutriaux C, Saiag P (2025). BO-112 plus pembrolizumab for patients with anti-PD-1-resistant advanced melanoma: phase II clinical trial SPOTLIGHT-203. J Clin Oncol.

